# Four-year follow up of macular intrachoroidal cavitation and chorioretinal atrophy: A case report

**DOI:** 10.1097/MD.0000000000028269

**Published:** 2021-12-17

**Authors:** Sanae Abe, Takeshi Iwase

**Affiliations:** Department of Ophthalmology, Akita University Graduate School of Medicine, Akita, Japan.

**Keywords:** chorioretinal atrophy, macular intrachoroidal cavitation, optical coherence tomography, pathologic myopia

## Abstract

**Rationale:**

Macular intrachoroidal cavitation (ICC) is characterized by presence of a hyporeflective space beneath the unaltered retinal pigment epithelium (RPE) and is noted around the region of focal chorioretinal atrophy in eyes with pathologic myopia. The findings suggest that the patchy chorioretinal atrophy (PCA) progresses to ICC with time. However, there have been no reports describing long-term observational studies using OCT. We collected a case of PCA progression to macular ICC using OCT during a 4-year follow-up.

**Patient concerns:**

A 65-year-old woman presented with metamorphopsia and blurred vision in her left eye. Her best-corrected visual acuity (BCVA) was 20/20 and spherical equivalent refraction was –14.0 diopters in the left eye. Fundus examination revealed a white, well-defined PCA at the superonasal to the fovea which showed hypofluorescence determined by autofluorescence in the left eye. Sclera curved posteriorly at the superonasal to the fovea and the choroid was thickened at the area and ellipsoid zone (EZ) was disrupted in the area in OCT images. Additionally, another OCT images through the PCA showed a disappearance of the RPE–Bruch's membrane complex and a connection of blood vessels running from the sclera to the choroid.

**Diagnoses:**

PCA with macular ICC.

**Interventions:**

Observation.

**Outcomes:**

During 4-year follow up, the white patchy lesion and the hypofluorescence region gradually expanded. BCVA decreased with the expansion of the lesion and was 20/100 at the final visit. OCT through the fovea showed that the disorganized EZ expanded toward the ICC and the sensory retina of the fovea became thinner. Moreover, RPE–Bruch's membrane complex was not observed 3 years after the initial visit. During the follow-up period, the sensory retina was prominently displaced posteriorly to the ICC, though no obvious change was observed in the structure of the sclera.

**Lessons:**

In cases of PCA with macular ICC, the outer retina and RPE may initially atrophy, showing as an expansion of PCA, because the attachment between the inner retina and sclera may be weakened. This may result in the displacement of the retinal tissue into the space of macular ICC.

## 1 Introduction

Pathological myopia is one of major cause of vision loss and legal blindness worldwide, with high incidence in East Asian countries.^[1,2]^ The fundus changes, characteristic of pathological myopia, occur at the macula due to the progressive axial elongation of the globe, the presence of posterior staphyloma, and the advancement of age. The vision loss in pathological myopia is mainly caused by the presence of myopic choroidal neovascularization, macular retinoschisis, and choroidal atrophy.^[3]^ Optical coherence tomography (OCT) is a useful tool in assessing those changes associated with fundic myopia progression.^[4]^

Freund et al first described intrachoroidal cavitation (ICC) in the peripapillary area as a “peripapillary detachment” and described it as an elevated, orange-yellow lesion at the inferior border of the myopic conus.^[5]^ ICC is a morphological feature, outlined by OCT, characterized by the presence of a hyporeflective space beneath the unaltered retinal pigment epithelium (RPE). ICC lesions are found at one or more locations, including the macula, the inferior, or temporal peripapillary disc. There are two types of myopic chorioretinal atrophies: diffuse chorioretinal atrophy and patchy chorioretinal atrophy (PCA).^[1,3,6–8]^

Ohno-Matsui et al reported 31 of 56 eyes (55.4%) with PCA showed macular ICC, using swept source OCT. Macular ICC is associated with posterior staphyloma and pathologic myopia and is noted around the region of focal chorioretinal atrophy. And the characteristic of macular ICC was similar to peripapillary ICC.^[2]^ The findings suggest that the PCA progresses to ICC with time and further progression of myopia. However, there have been no reports describing long-term observational studies about the relationship between macular ICC and the progression of PCA. This highlights the need for such studies to confirm this hypothesis. We collected a case of PCA progression to macular ICC using OCT during a 4-year follow-up.

## 2 Case report

**Figure 1:**
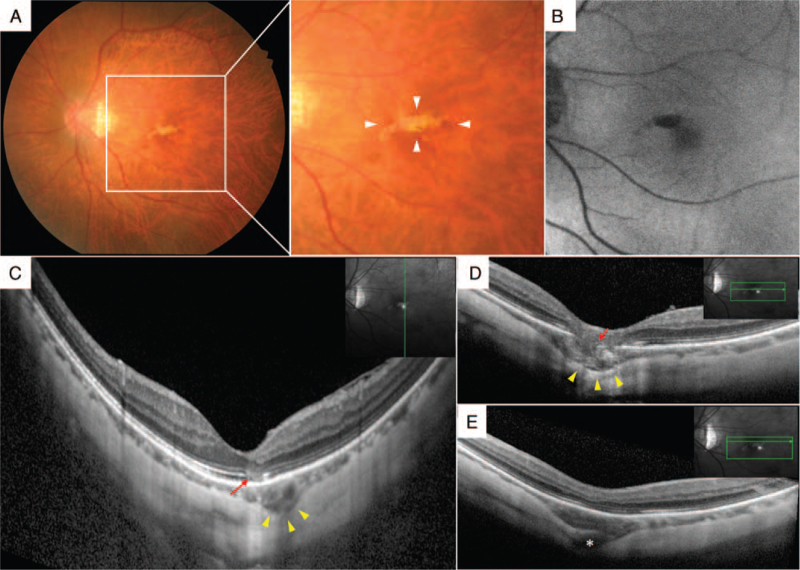
Initial visit findings in the left eye. Color fundus showed a white well-defined patchy lesion at the superonasal to the fovea (A), and autofluorescence showed hypofluorescence area on the lesion (B). OCT showed that the sclera curved posteriorly at the location superonasal to the fovea, the choroid was thickened, and EZ was disrupted in the area (yellow arrow head) in the vertical section through the fovea (C). Horizontal volume scans of the OCT through the patchy lesion showed posterior curvature of the sclera, thinning of the outer retina, and disappearance of the RPE–Bruch's membrane complex (D). Another horizontal volume scan of the OCT demonstrated a connection of blood vessels running from the sclera (asterisk) to the choroid (E).

A 65-year-old woman presented with metamorphopsia and blurred vision in her left eye and was referred to Akita City Hospital for further examination. She had hypertension and dyslipidemia, which were treated with oral medication. At the initial visit, her best-corrected visual acuity (BCVA) was 20/20 in both eyes. Spherical equivalent refraction was –10.5 diopters and –14.0 diopters in the right and left eyes, respectively. The intraocular pressure was 18 and 16 mm Hg in the right and left eyes, respectively. Slit-lamp examination showed a slight nuclear cataract of grade 1 in both eyes, based on Emery-Little classification. Fundus examination revealed tigroid fundus in both eyes, and a white, well-defined, and patchy lesion at the superonasal to the fovea in the left eye (Fig. 1A). Autofluorescence showed hypofluorescence area in the macula. This is consistent with the aforementioned lesion in the left eye (Fig. 1B).

Upon applying OCT to the vertical section through the fovea, the sclera curved posteriorly at the location superonasal to the fovea, and the choroid was thickened in the area (Fig. 1C). Additionally, the ellipsoid zone (EZ) was disrupted in the area between the fovea and the curved sclera. Horizontal volume scans of the OCT through the patchy lesion also showed posterior curvature of the sclera, thinning of the outer retina, and disappearance of the RPE–Bruch's membrane complex, resulting in a pseudo-fovea appearance (Fig. 1D). Another horizontal volume scan of the OCT demonstrated a connection of blood vessels running from the sclera to the choroid (Fig. 1E). There was no obvious connection between the vitreous cavity and the superior choroidal cavity with OCT; taken together, we diagnosed this case as PCA with macular ICC.

**Figure 2:**
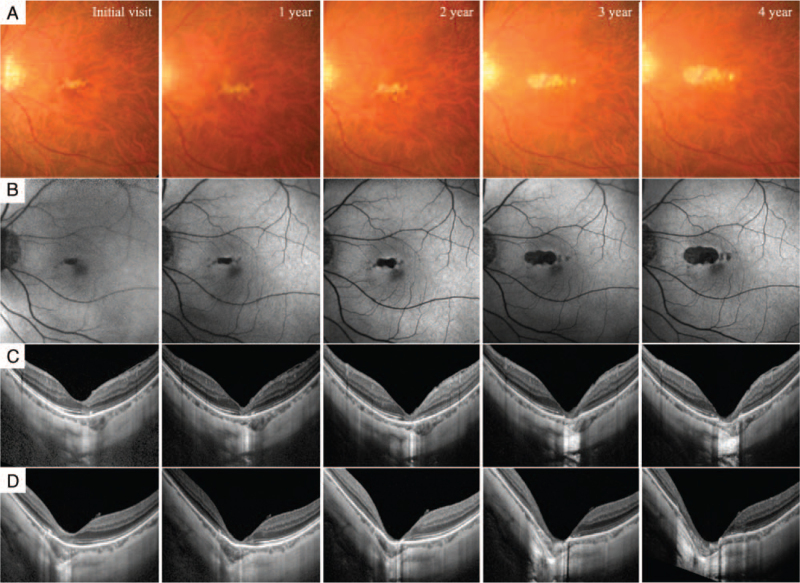
Change of PCA during a 4-year follow up. The white patchy lesion in the fundus (A) and the hypofluorescence region determined by autofluorescence (B) gradually expanded, especially toward the optic disc. OCT through the fovea showed that the disorganized EZ expanded toward the ICC and the sensory retina of the fovea became thinner with time (C). RPE–Bruch's membrane complex was not observed 3 years after the initial visit (D). The sensory retina was prominently displaced posteriorly to the ICC, though no obvious change was observed in the structure of the sclera.

During a 4-year follow up, the white patchy lesion in the fundus and the hypofluorescence region determined by autofluorescence gradually expanded, especially toward the optic disc (Fig. 2A and B). The BCVA in the left eye decreased with the expansion of the lesion and was 20/100 at the final visit. OCT through the fovea showed that the disrupted EZ expanded toward the ICC and the sensory retina of the fovea became thinner with time (Fig. 2C). Moreover, RPE–Bruch's membrane complex was not observed 3 years after the initial visit. During the follow-up period, the sensory retina was prominently displaced posteriorly to the ICC, though no obvious change was observed in the structure of the sclera.

## 3 Discussion

Macular ICC was reported to be found in 31 of 56 PCA eyes (55.4%), which were in the macular area and surrounding the PCA.^[3]^ However, the pathology of ICC remains unknown as well as the relationship between the ICC and the progression of PCA. Here, we report a long-term clinical course of PCA progression with macular ICC. Our results show that, over the course of 4 years, the PCA gradually enlarged along with retinochoroidal morphological changes determined by OCT. However, the structure of the ICC did not change.

OCT imaging showed that the sclera was curved backward from the fovea to the superonasal area. OCT also revealed that the choroid in the area was thicker than that surrounding the ICC. During the 4-year follow-up period, the white patchy lesion and the hypofluorescent area enlarged, which resulted in foveal involvement of the PCA. Consistent with these findings, OCT showed gradual expansion of the disorganized EZ and disappearance of the RPE in the area. Additionally, the retina thinned, which resulted in deviation into the ICC. PCA severely impacts vision due to the absence of underlying choroid and the degeneration of overlying RPE and photoreceptors.^[7,9]^ The progression and expansion of these lesions is the leading cause of vision loss in pathological myopia.^[6]^ Consequently, BCVA decreased from 20/20 to 20/100 during the follow-up period in our case.

Various speculations have come forth regarding the occurrence of macular ICC. Venkatesh et al suggest that as the posterior staphyloma progresses, the tissue at the edge of the PCA may be stretched and destroyed, allowing vitreous fluid to gain access to the suprachoroidal space, which causes macular ICC.^[3]^ In our case, however, OCT showed no apparent connection between the vitreous cavity and the superior choroidal cavity.

Ohno-Matsui et al suggest that the ocular wall is thinner and more susceptible to IOP than the normal area away from the PCA since the PCA area lacks the retinal and choroidal layers.^[3]^ Therefore, expansion of the posterior wall should be more pronounced. Additionally, the sclera in the area adjacent to the intrascleral vessels is less elastic. The presence of PCA in that area may result in increased susceptibility to the effects of elevated IOP.^[2]^ In our case, the PCA gradually enlarged over 4 years though the size of the macular ICC did not change. These findings suggest that, contrary to previous reports, macular ICC might facilitate the dissolution of retinal tissue, especially RPE and the outer retina, in the area where the attachment between the inner retina and sclera might be weak. This, along with posterior scleral bowing, might be responsible for the displacement of retinal tissue into the space of cavitation.

## 4 Conclusion

In cases of PCA with macular ICC, the outer retina and RPE may initially atrophy, showing as an expansion of PCA, because the attachment between the inner retina and sclera may be weakened. This may result in the displacement of the retinal tissue into the space of macular ICC.

## Author contributions

**Conceptualization:** Sanae Abe, Takeshi Iwase.

**Data curation:** Sanae Abe.

**Funding acquisition:** Takeshi Iwase.

**Methodology:** Sanae Abe, Takeshi Iwase.

**Supervision:** Takeshi Iwase.

**Validation:** Sanae Abe, Takeshi Iwase.

**Visualization:** Takeshi Iwase.

**Writing – original draft:** Sanae Abe, Takeshi Iwase.

**Writing – review & editing:** Takeshi Iwase.
